# Supplementary material for: Assessing the impact of Enhanced-Case-Finding on tuberculosis case notifications and transmission in The Gambia using epidemiological and phylodynamic approaches
Source: Open Res Eur. 2026 Apr 10;6:96. [Version 1] doi: 10.12688/openreseurope.21214.1 (PMC13157611; doi:10.12688/openreseurope.21214.1)
Supplement: Peer review history

# Reviewer report: Reviewer response for version 1

**DOI:** 10.21956/openreseurope.22947.r72233

**Published:** 2026-05-09

**Authors:** Benjamin Sobkowiak

**Affiliations:** 1 University College London, London, UK

Here, the authors present a cluster randomized trial evaluating the impact of enhanced case finding on TB case notifications and transmission in The Gambia, integrating epidemiological modelling with genomic and phylodynamic analyses.

 The study is generally strong and is an innovative design due to its combination of classical epidemiological trial design with whole genome sequencing and phylodynamic modelling to assess transmission dynamics, which despite the null findings with ECF found in the study, can be a framework for future work. Additionally, the use of multiple complementary transmission metrics (case notification rates, SNP clustering, and Re estimates) provides multiple metrics to evaluate the impact of intervention. I feel the paper could benefit from some small, additional details to improve the manuscript.

 Comments:

 - Phylodynamic methods - The phylodynamic modelling framework is technically rigorous but would benefit from a clearer description of key parameters (e.g., *Re* and the intervention effect parameter f), justification of priors, and confirmation of any temporal signal before the phylodynamic analysis.

 - Genomic clusters - The number and proportion of SNP-based transmission clusters is reported but not the distribution of cluster sizes (e.g., median size, range, or frequency of large clusters). Providing this information would help interpret transmission dynamics and assess whether a small number of large clusters or many small clusters drive transmission in this setting, as well as potentially highlighting the impact of the sampling density on the results.

 - Interpretation – The paper might benefit from further discussion about the null finding in the context of the sampling density and intervention intensity. In particular, the relatively low proportion of sequened cases may have reduced the study’s ability to detect differences in transmission dynamics between arms of the trial. While there is some discussion in the limitation, the authors could strengthen this section by linking diagnostic and sampling constraints to the interpretability of transmission metrics (e.g., clustering and *Re*), and discussing how they may have reduced the ability to detect an intervention effect.

Is the study design appropriate and does the work have academic merit?

Yes

Is the work clearly and accurately presented and does it cite the current literature?

Yes

If applicable, is the statistical analysis and its interpretation appropriate?

Yes

Are all the source data underlying the results available to ensure full reproducibility?

Yes

Are the conclusions drawn adequately supported by the results?

Yes

Are sufficient details of methods and analysis provided to allow replication by others?

Yes

Reviewer Expertise:

Bioinformatics, genomic epidemiology

I confirm that I have read this submission and believe that I have an appropriate level of expertise to confirm that it is of an acceptable scientific standard, however I have significant reservations, as outlined above.

# Reviewer report: Reviewer response for version 1

**DOI:** 10.21956/openreseurope.22947.r72238

**Published:** 2026-05-07

**Authors:** André C Pereira

**Affiliations:** 1 Instituto Superior Técnico, Lisboa, Portugal

The manuscript presents a well-structured and coherent investigation into the impact of enhanced case finding (ECF) on tuberculosis notification and transmission in The Gambia. The integration of classical epidemiological approaches with genomic and phylodynamic analyses is a notable strength, providing a comprehensive framework to assess intervention outcomes.

 Minor comments:

 - Authors use “people living with TB” repeatedly; consider replacing with “TB patients” or “individuals diagnosed with TB” for clarity and consistency with standard terminology.

 - In the "methods" section, the "Phylogenetic Analyses" subsection is a strong and sophisticated component; however, the description is mathematically dense and may be difficult for a general audience. Please consider adding a brief intuitive explanation of the model assumptions.

 The same applies to the “Prior Distributions” and “Sensitivity Analyses” sections. Please better contextualize the chosen assumptions and parameter settings, and clarify their roles in the overall analyses.

 - Please add the rationale for the absence of a temporal signal analysis prior to the phylodynamic inference, since ensuring a temporal signal in data is essential for a reliable phylodynamic inference.

 - Table 1 is misplaced and mislinked to Supplementary Table 1 in the methods section; please rectify.

 - Several typos can be found throughout the manuscript; please revise carefully.

 - Please improve the quality of Figure 3.

 - There is ambiguity regarding the inclusion of minors. While participants aged 15–17 are reported as included with appropriate consent procedures, it is later stated that individuals under 18 were excluded from certain analyses. This discrepancy should be clearly explained, especially by distinguishing between participation in the clinical study and inclusion in downstream genomic analyses.

 - The description of consent procedures for the community-level intervention would also benefit from further elaboration. Although individual consent may not be required in such cluster-based interventions, it would be important to explicitly state how community approval was obtained and justify this approach from an ethical perspective.

 - Please further discuss the results in light of previous TB reports in Gambia (e.g., 10.2471/BLT.14.151670, 10.1086/591504, 10.1186/s12889-020-09685-3, 10.1186/1471-2458-6-156, 10.1016/j.ijid.2020.09.029, 10.1038/s41598-026-42003-2, 10.1128/JCM.01216-08)

Is the study design appropriate and does the work have academic merit?

Yes

Is the work clearly and accurately presented and does it cite the current literature?

Partly

If applicable, is the statistical analysis and its interpretation appropriate?

Yes

Are all the source data underlying the results available to ensure full reproducibility?

Yes

Are the conclusions drawn adequately supported by the results?

Yes

Are sufficient details of methods and analysis provided to allow replication by others?

Yes

Reviewer Expertise:

Animal infectious diseases; Molecular epidemiology; Phylodynamic analyses; Microbiome

I confirm that I have read this submission and believe that I have an appropriate level of expertise to confirm that it is of an acceptable scientific standard.

## References

[1] : A tuberculosis nationwide prevalence survey in Gambia, 2012. *Bulletin of the World Health Organization* .2016;94(6) : 10.2471/BLT.14.151670 433-441 10.2471/BLT.14.151670 27274595 PMC4890202

[2] : Progression to Active Tuberculosis, but Not Transmission, Varies byMycobacterium tuberculosis Lineage in The Gambia. *The Journal of Infectious Diseases* .2008;198(7) : 10.1086/591504 1037-1043 10.1086/591504 18702608 PMC2597014

[3] : Knowledge, attitude and practice towards tuberculosis in Gambia: a nation-wide cross-sectional survey. *BMC Public Health* .2020;20(1) : 10.1186/s12889-020-09685-3 10.1186/s12889-020-09685-3 33069220 PMC7568354

[4] : Risk factors for pulmonary tuberculosis: a clinic-based case control study in The Gambia. *BMC Public Health* .2006;6(1) : 10.1186/1471-2458-6-156 10.1186/1471-2458-6-156 16784521 PMC1543634

[5] : Delay in the diagnosis of pulmonary tuberculosis in The Gambia, West Africa: A cross-sectional study. *International Journal of Infectious Diseases* .2020;101: 10.1016/j.ijid.2020.09.029 102-106 10.1016/j.ijid.2020.09.029 PMC749372832949776

[6] : Genome-wide analyses of Mycobacterium tuberculosis complex isolates reveal insights into circulating lineages and drug resistance mutations in The Gambia. *Scientific Reports* .2026;16(1) : 10.1038/s41598-026-42003-2 10.1038/s41598-026-42003-2 PMC1306896641781593

[7] : Use of Spoligotyping and Large Sequence Polymorphisms To Study the Population Structure of theMycobacterium tuberculosis Complex in a Cohort Study of Consecutive Smear-Positive Tuberculosis Cases in The Gambia. *Journal of Clinical Microbiology* .2009;47(4) : 10.1128/JCM.01216-08 994-1001 10.1128/JCM.01216-08 PMC266836219193842

# Reviewer report: Reviewer response for version 1

**DOI:** 10.21956/openreseurope.22947.r72240

**Published:** 2026-05-07

**Authors:** Katharine S Walter

**Affiliations:** 1 The University of Utah, Salt Lake City, Utah, USA

Gehre et al. conducted a cluster randomized control trial to test the impact of enhanced-case-finding (ECF) on tuberculosis notification rates and transmission in a high-incidence setting in The Gambia. The intervention included community health worker-led education about TB symptoms and voluntary submission of self-sampled sputum from individuals for smear microscopy testing. Passive case finding continued in both the intervention and control arms. The study found no significant difference in TB case notification rates between study arms, nor differences in transmission metrics estimated with *M. tuberculosis *genomic data (SNP-based genomic clustering and the effective reproductive number). The authors discuss that this null finding could reflect the relatively short follow-up period of the study, or a limited effect of the intervention.

 There is a critical need to identify the estimated three million people with undiagnosed TB worldwide. While this study does not find evidence to support the ECF intervention studied here, the authors use an innovative genomic epidemiology approach to measure the effect of the intervention on TB transmission with genomic clustering proportion and the effective reproductive number.

 The integration of genomic and epidemiologic endpoints in an RCT context is an important contribution. Below are comments.

  

- The Abstract could be improved with more quantitative results, including case notification rates and proportion of genomes falling within SNP-based clusters in the intervention and control arms.
- Consider defining the term “sensitization” in the Abstract methods.
- The word cluster is used to describe both the intervention/control areas as well as the SNP-based transmission cluster. Perhaps using the term “transmission cluster” or “SNP-based cluster” could help distinguish this.
- Were socio-demographic characteristics of participants similar in the intervention and control arm?  
- Results: Please report the case notification rate for the control and intervention arms over the duration of the study period along with the difference in case notification rates shown in Fig. 3.
- Results: What proportion of the 150 SNP-based transmission clusters included individuals in both the control and intervention arms? This might indicate a degree of non-independence between the study arms.
- The Limitations section discussed the reach of the educational intervention. Is it known how many people participated in community-based education offerings?
- Does sputum transport impact microscopy test sensitivity?
- Could relatively low proportion of culture-positive samples (43%) limit the power of genomic measures to detect an effect of the intervention on measures of transmission?
- Is there a reference for the finding that the MTP64-based rapid test has poor sensitivity for detection of Mtb L5 and L6?

Is the study design appropriate and does the work have academic merit?

Yes

Is the work clearly and accurately presented and does it cite the current literature?

Yes

If applicable, is the statistical analysis and its interpretation appropriate?

Yes

Are all the source data underlying the results available to ensure full reproducibility?

Yes

Are the conclusions drawn adequately supported by the results?

Yes

Are sufficient details of methods and analysis provided to allow replication by others?

Yes

Reviewer Expertise:

Epidemiology

I confirm that I have read this submission and believe that I have an appropriate level of expertise to confirm that it is of an acceptable scientific standard, however I have significant reservations, as outlined above.
